# Smoking prevalence among hospitalized COVID-19 patients and its association with disease severity and mortality: an expanded re-analysis of a recent publication

**DOI:** 10.1186/s12954-020-00437-5

**Published:** 2021-01-16

**Authors:** Konstantinos Farsalinos, Pantelis G. Bagos, Theodoros Giannouchos, Raymond Niaura, Anastasia Barbouni, Konstantinos Poulas

**Affiliations:** 1 Laboratory of Molecular Biology and Immunology, Department of Pharmacy, University of Patras, 26500 Rio-Patras, Greece; 2 School of Public Health, University of West Attica, Leoforos Alexandras 196A, 11521 Athens, Greece; 3 Department of Computer Science and Biomedical Informatics, University of Thessaly, 35100 Lamia, Greece; 4 Pharmacotherapy Outcomes Research Center, College of Pharmacy, University of Utah, Salt Lake City, USA; 5 Laboratory of Health Economics and Management, Economics Department, University of Piraeus, Piraeus, Greece; 6 Departments of Social and Behavioral Science and Epidemiology, College of Global Public Health, New York University, New York City, USA

## Abstract

**Background:**

There is a lot of debate about the effects of smoking on COVID-19. A recent fixed-effects meta-analysis found smoking to be associated with disease severity among hospitalized patients, but other studies report an unusually low prevalence of smoking among hospitalized patients. The purpose of this study was to expand the analysis by calculating the prevalence odds ratio (POR) of smoking among hospitalized COVID-19 patients, while the association between smoking and disease severity and mortality was examined by random-effects meta-analyses considering the highly heterogeneous study populations.

**Methods:**

The same studies as examined in the previous meta-analysis were analyzed (*N* = 22, 20 studies from China and 2 from USA). The POR relative to the expected smoking prevalence was calculated using gender and age-adjusted population smoking rates. Random-effects meta-analyses were used for all other associations.

**Results:**

A total of 7162 patients were included, with 482 being smokers. The POR was 0.24 (95%CI 0.19–0.30). Unlike the original study, the association between smoking and disease severity was not statistically significant using random-effects meta-analysis (OR 1.40, 95%CI 0.98–1.98). In agreement with the original study, no statistically significant association was found between smoking and mortality (OR 1.86, 95%CI 0.88–3.94).

**Conclusion:**

An unusually low prevalence of smoking, approximately 1/4th the expected prevalence, was observed among hospitalized COVID-19 patients. Any association between smoking and COVID-19 severity cannot be generalized but should refer to the seemingly low proportion of smokers who develop severe COVID-19 that requires hospitalization. Smokers should be advised to quit due to long-term health risks, but pharmaceutical nicotine or other nicotinic cholinergic agonists should be explored as potential therapeutic options, based on a recently presented hypothesis.

## Introduction

The association between smoking and COVID-19 has generated a lot of interest in the research community. Smoking is an established risk factor for respiratory infections [1]. Therefore, it was not surprising that reports suggested a higher risk for severe COVID-19 among hospitalized smokers [2–4]. However, these studies failed to notice the relatively low prevalence of smoking among hospitalized patients compared to population smoking rates [5, 6]. This was first noticed in Chinese case series, but similar findings have been observed in other countries, while it has also been reported that smoking may be associated with lower susceptibility for SARS-CoV-2 infection [7–10]. The possibility that smokers may be less likely to develop severe COVID-19 that would require hospitalization is an important factor in determining the overall smoking-related risk. A higher risk for adverse outcome among hospitalized smokers is not applicable to all smokers if they are indeed less likely than non-smokers to be hospitalized for COVID-19. In March, we hypothesized for the first time that nicotine may be protective against COVID-19 due to its anti-inflammatory properties and to a potential direct interaction between SARS-CoV-2 and nicotinic acetylcholine receptors [11, 12]. The cholinergic anti-inflammatory pathway represents a reflex mechanism that modulates the immune response and protects from hyper-inflammation, a hall mark of severe COVID-19 [13, 14]. Therefore, if the virus interacts with the cholinergic system, dysregulation of the cholinergic anti-inflammatory pathway could result in an uncontrolled immune response. This hypothesis is not contradictory to reports of a higher risk for adverse outcome in hospitalized smokers with COVID-19. Smokers experience abrupt cessation of nicotine intake once hospitalized (unless nicotine replacement therapies are administered), resulting in the rapid elimination of plasma nicotine levels and deprivation of any hypothetical beneficial effects.

Recently, Karanasos et al. [15] published a systematic review and meta-analysis of 22 studies, examining the impact of smoking on disease severity and mortality of hospitalized patients with COVID-19 infection. They also performed a meta-regression analysis and stratified studies according to the prevalence of diabetes among patients (< 15% and ≥ 15%). They reported that smoking was associated with higher odds of disease severity in studies with low prevalence of diabetes. However, the authors did not examine the smoking prevalence among hospitalized COVID-19 patients relative to the population smoking rates. Additionally, we noticed minor errors in the data presented (mentioned below) which were addressed in the present analysis. Finally, the authors used a fixed-effects method for the meta-analysis. This is rather odd and probably inappropriate, especially when it comes to the justification they proposed. The authors stated that they used fixed effects due to non-significant heterogeneity (*I*^2^ < 50%). This particular approach is questionable since the *I*^2^ purpose is to quantify the degree of heterogeneity and not to test its significance. Indeed, the respective statistical test based on the Cochran’s chi-square yielded a significant *p* value of 0.02. (Note that this test has low power and thus a significant result is even more important). That is, if the authors were to choose based on purely statistical arguments, they should have chosen the random-effects model. Nevertheless, the choice of fixed vs. random effects has been a matter of debate in the literature and the prevailing approach is that the model choice for meta-analysis should be based on the sampling frame and not on the results of a statistical test such as the test for heterogeneity in effect sizes [16, 17]. The studies analyzed included patients from different hospitals, geographical locations and countries, and age and comorbidities. Additionally, even the definition of smoking was not universal in all studies, with some reporting current and former smoking while others reporting “smoking history” or “smoking” [13]. Taking into account that the primary goal of such an analysis is to generalize the results, one would argue that the random-effects model should have been the method of choice in the first place, irrespective of the identified heterogeneity [16]. For these reasons, the random-effects model is considered more appropriate and is advocated by most experts [16, 18, 19]. We also need to emphasize that in case of zero heterogeneity the estimates of both models coincide. Moreover, the choice of the fixed-effects model comes to a direct disagreement with the subsequent use of a random-effects meta-regression performed by the authors [15]. Meta-regression is used to explore the sources of heterogeneity [20]; thus, its use contradicts the initial argument for choosing the fixed-effects model.

Considering the above, and to address potential errors in the original study, we re-analyzed the data and expanded the analysis by: (1) calculating the prevalence odds ratio (POR) [21] of smoking among hospitalized COVID-19 patients relative to population smoking rates and (2) examining the association between smoking and COVID-19 severity and mortality, as well as the association between smoking and severity with studies stratified according to diabetes prevalence (< 15% and ≥ 15%).

## Methods

The same studies as examined by Karanasos et al. were analyzed herein (*N* = 22) [22–43]. Data extraction was performed by two authors (K.F., P.B.). Smoking prevalence was derived from the tables of each publication. Similarly to Karanasos et al., the studies were stratified by country in two groups: China and USA. The following minor errors were noticed in the original analysis: (1) In the study by Shi et al. [34], 434 patients were non-smokers (487 patients in total, of whom 40 were smokers, 434 were non-smokers, and 13 had unknown smoking history). Karanasos et al. presented 433 non-smokers in the original analysis. (2) In the study by Chen et al. [40], 12 patients were current smokers and seven were former smokers. The original analysis by Karanasos et al. included former smokers as current smokers, but in all other studies former smokers were not included into the current smokers group. 3. In the study by Wang et al. [41], two groups of different patients were presented, both of which had data on survival and smoking status (*n* = 296 and *n* = 44). Only the first group was included in the original analysis, while we included all patients in the current analysis.

Smoking prevalence in each study was compared with the expected prevalence based on gender and age-adjusted population smoking rates. The gender distribution of patients (proportion of males and females) in each study was used for the gender adjustment. No gender adjustment was performed for one study because of unavailable data [38]. Since no data were available on the age distribution of patients, age adjustment was performed by assuming that all patients were aged ≥ 65 years. This age group has the lowest smoking rates in both China and the USA compared to other adult age groups, while the mean or median age of patients in the studies was lower than 65 (Table 1). Thus, this age-adjustment underestimates the expected smoking prevalence. The following formula was used to calculate the expected smoking prevalence:

$${\text{SP}}_{{\text{E}}} = \, \left( {P_{{\text{M}}} x{\text{ SP}}_{{{\text{P}} - {\text{M}}}} } \right) \, + \, \left( {{\text{P}}_{{\text{F}}} x{\text{ SP}}_{{{\text{P}} - {\text{F}}}} } \right)$$

where SP_E_ = expected smoking prevalence; *P*_M_ = male prevalence among patients; SP_P–M_ = population smoking prevalence in males ≥ 65 years old; *P*_F_ = female prevalence among patients; SPP-F = population smoking prevalence in females ≥ 65 years old.

**Table 1:** Characteristics of the studies included in the analysis, and gender and age-adjusted expected smoking prevalence based on population smoking rates

	Hospitalized cases	Age	Males	Females	Hospitalized smokers	Hospitalized smokers prevalence	Expected number of smokers (gender and age-adjusted)	Expected smokers gender and age-adjusted
	*N*	Median (IQR) Mean (SD)	%	%	*n*	% (95%CI)	*n*	%
Chen et al. [22]	145	48 (15)	54.5	45.5	15	10.3 (5.9–16.5)	37	25.8
Feng et al. [23]	454	53 (60–64)	56.9	43.1	44	9.7 (7.1–12.8)	122	26.8
Guan et al. [24]	1085	47 (35–58)	58.1	41.9	137	12.6 (10.6–14.6)	296	27.3
Hu et al. [25]	323	61 (23–91)	51.4	48.6	38	11.8 (8.5–15.8)	79	24.6
Huang et al. [26]	41	49 (41–58)	73.2	26.8	3	7.3 (0.0–15.3)	14	33.3
Ji et al. [27]	208	44 (16)	56.3	43.8	19	9.2 (5.6–13.9)	55	26.5
Li et al. [28]	544	60 (48–69)	51.3	48.7	41	7.5 (5.4–10.1)	134	24.6
Li et al. [29]	25	51	48.0	52.0	7	28.0 (12.1–49.4)	6	23.3
Liu et al. [30]	40	49 (14)	37.5	62.5	5	12.5 (4.2–26.8)	8	19.1
Liu et al. [31]	78	38 (33–57)	50.0	50.0	5	6.4 (0.1–11.8)	19	24.1
Mo et al. [32]	155	54 (42–66)	55.5	44.5	6	3.9 (0.9–6.9)	41	26.2
Qin et al. [33]	452	58 (47–67)	52.0	48.0	7	1.6 (0.6–3.2)	112	24.8
Shi et al. [34]	474	46 (19)	53.2	46.8	40	8.4 (6.1–11.3)	120	25.3
Wan et al. [35]	135	47 (36–55)	53.3	46.7	9	6.7 (2.5–10.9)	34	25.4
Wang et al. [36]	125	39 (14)	56.8	43.2	16	12.8 (7.5–20.0)	33	26.8
Zhang et al. [37]	140	57 (25–87)	50.7	49.3	2	1.4 (0.0–3.3)	34	24.3
Chen et al. [38]	274	62 (44–70)	62.4	37.6	12	5.4 (2.4–8.3)	79	29.0
Wang et al. [39] (1)	340	47 (15) 55 (17)	48.2	51.8	16	4.7 (2.7–7.5)	79	23.3
Yang et al. [40]	52	52 (13)	67.3	32.7	2	3.8 (0.5–13.2)	16	31.0
Zhou et al. [41]	191	56 (46–67)	62.3	37.7	11	5.8 (2.5–9.1)	55	29.0
Chow et al. [42] (2)	1494				27	1.8 (1.2–2.6)	131	8.8
Goyal et al. [43]	393	62 (49–74)	60.6	39.4	20	5.1 (3.1–7.8)	36	9.2
Total	7168				482	7.0 (5.1–9.3)	1542	24.1 (20.1–28.3)

(1) Age was reported separately for the two groups of patients

(2) No data on age and gender were available. The expected smoking prevalence was not adjusted for gender

The population smoking rates used to calculate the age and gender-adjusted expected number of smokers for males and females in each study were 44.0% and 4.1% for China [44] and 10.1% and 7.7% for USA [45], respectively. The association between observed and expected smoking prevalence was measured by calculating the POR [21].

Eighteen studies were used to examine the association between smoking and COVID-19 severity [22–39]. For mortality, five studies were analyzed. One study included data on both disease severity and mortality, and it was used in both analyses [29]. The analysis of disease severity stratified by diabetes prevalence included ten studies with prevalence < 15% [22–25, 29, 31, 32, 34, 35, 37] and six studies with prevalence ≥ 15% [26, 28, 30, 33, 38, 39].

All analyses were performed with inverse variance random-effects meta-analyses using Review Manager (RevMan) 5.4 (Copenhagen: The Nordic Cochrane Centre, The Cochrane Collaboration, 2014).

## Results

The characteristics of the studies are presented in Table 1. The number of expected smokers based on gender and age-adjusted population smoking rates is also reported. In total, 7168 patients were analyzed, with 482 of them being smokers. The random-effects pooled prevalence of smoking was 7.0% (95%CI 5.1–9.3%). The expected pooled prevalence of smoking was calculated at 24.1% (95%CI 20.1–28.3%). Only one study had more smokers than the expected number of smokers [29].

**Fig. 1:**
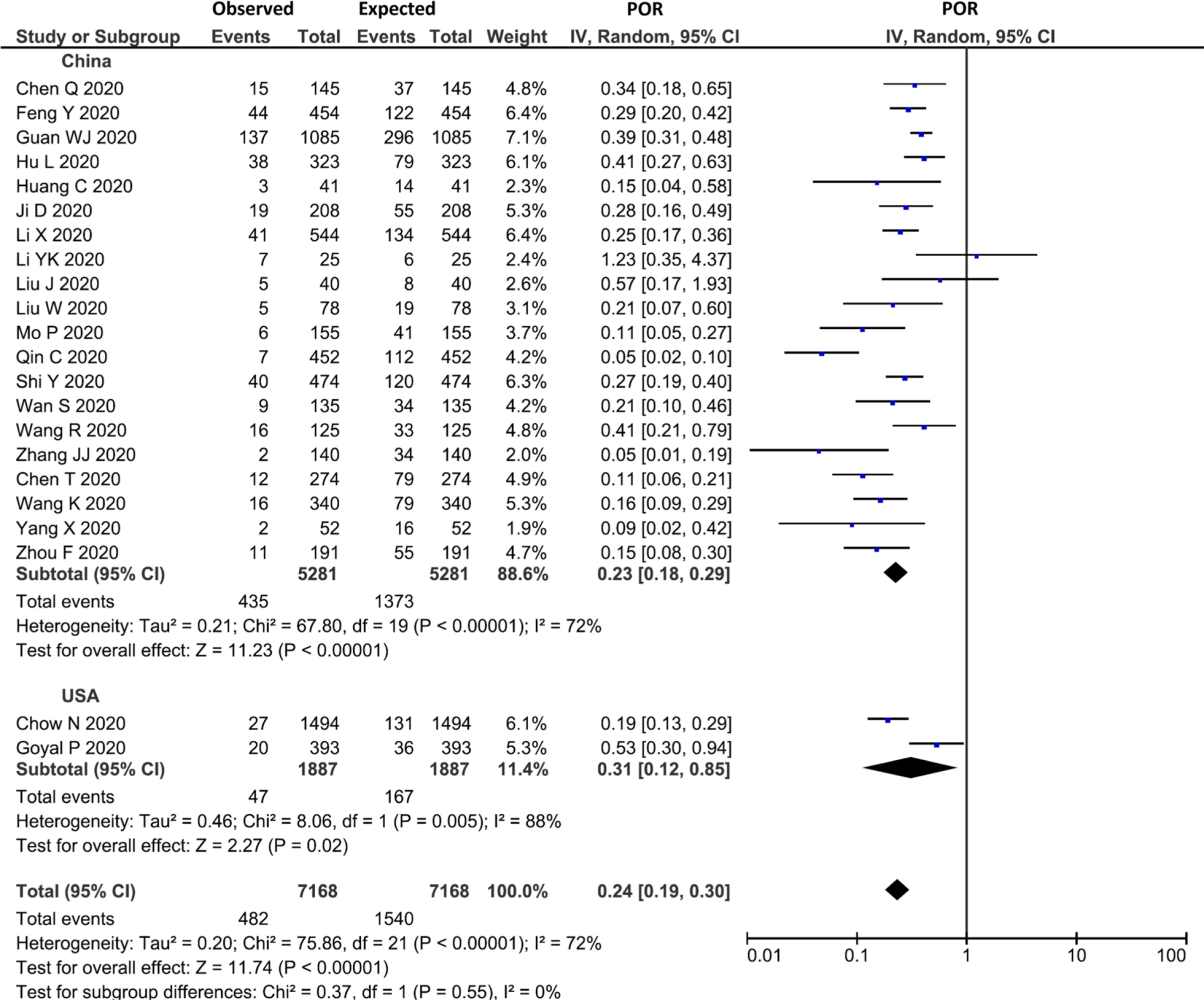
Prevalence odds ratio (POR) of smoking among hospitalized COVID-19 patients. Calculations were made by comparing the observed smoking prevalence with the expected prevalence, based on gender and age-adjusted population smoking rates, using random-effects meta-analysis. Boxes represent odds ratios (ORs) and lines represent the 95%CI

**Fig. 2:**
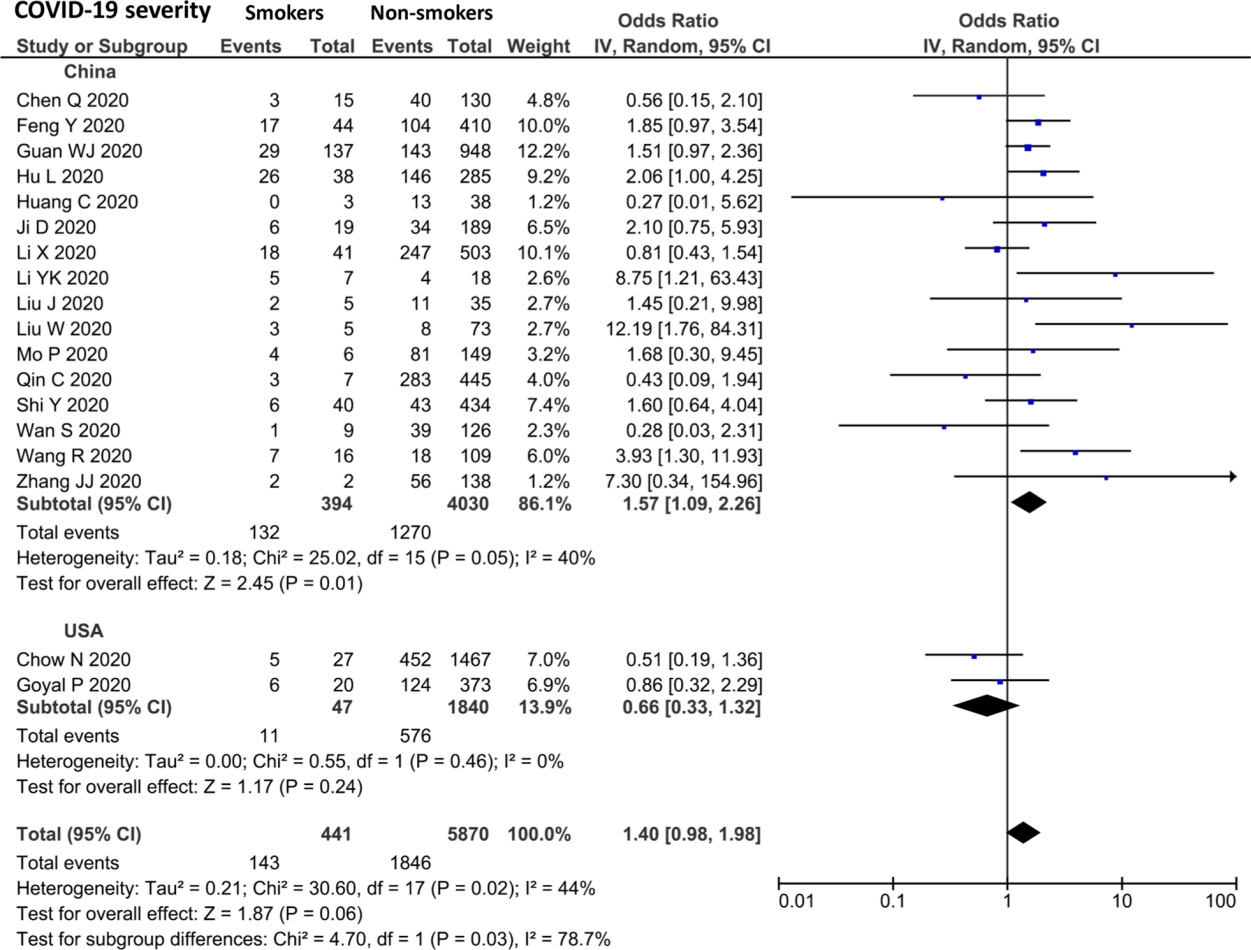
Random-effects meta-analysis of the association between smoking and COVID-19 severity. Boxes represent odds ratios (ORs) and their size represent the studies weight, while lines represent the 95%CI

**Fig. 3:**
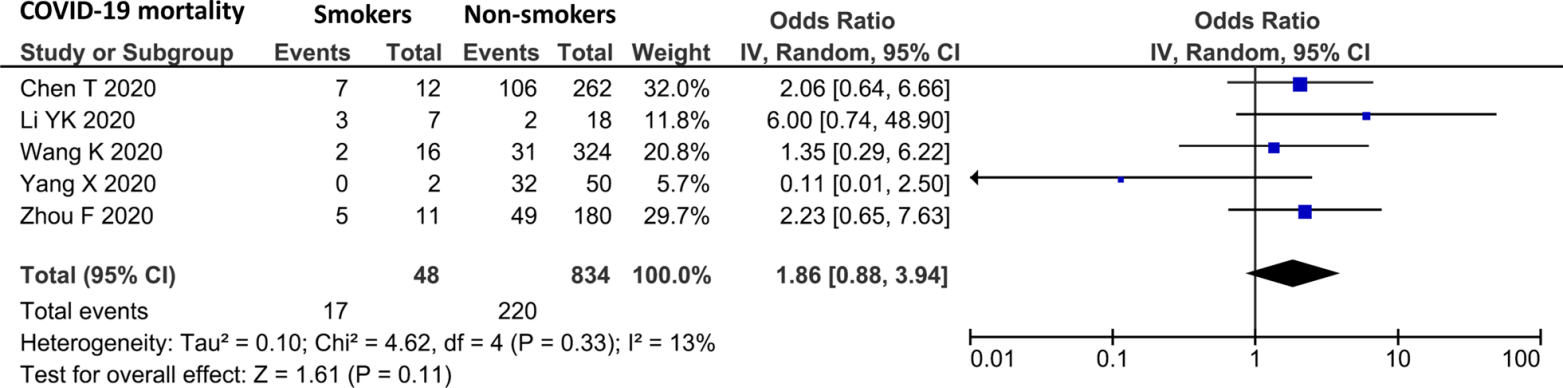
Random-effects meta-analysis of the association between smoking and COVID-19 mortality. Boxes represent odds ratios (ORs) and their size represent the studies weight, while lines represent the 95%CI

**Fig. 4:**
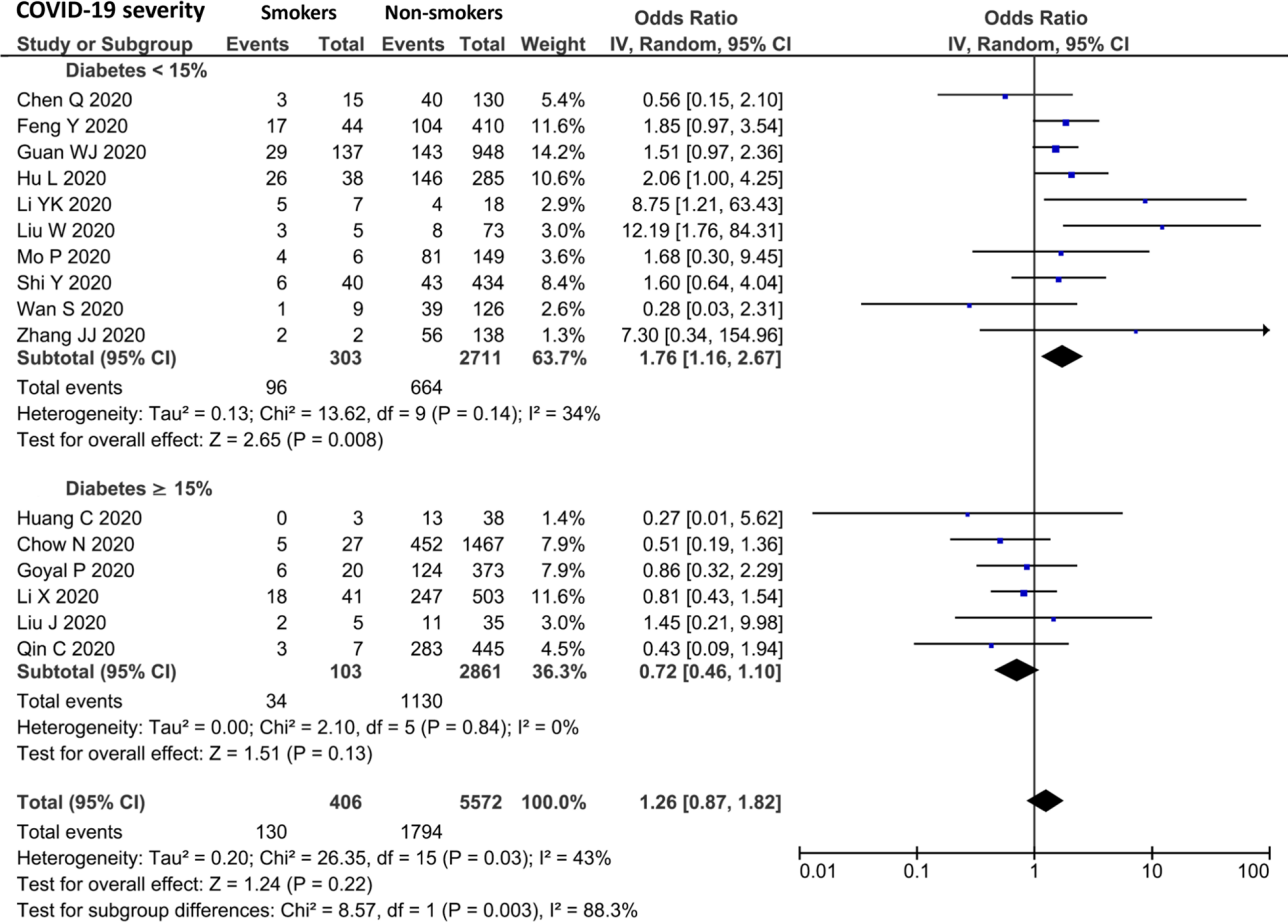
Random-effects meta-analysis of the association between smoking and COVID-19 severity with studies stratified by diabetes prevalence (< 15% and ≥ 15%). Boxes represent odds ratios (ORs) and their size represent the studies weight, while lines represent the 95%CI

The POR of smoking is presented in Fig. 1. The proportion of hospitalized COVID-19 patients who reported being smokers was approximately 1/4th and 1/3rd the expected proportion based on population smoking rates in China and USA, respectively, and 1/4th in the total sample. This indicates a substantial under-representation of smokers. The results of the random-effects meta-analysis of the association between smoking and disease severity and mortality are presented in Figs. 2 and 3, respectively. A statistically significant association was observed between smoking and disease severity in the Chinese studies (OR 1.57, 95%CI 1.09–2.26) but not in the US studies (OR 0.66, 95%CI 0.33–1.32). No statistically significant association between smoking and COVID-19 severity was found when all studies were analyzed (OR 1.40, 95%CI 0.98–1.98). No statistically significant association was observed between smoking and COVID-19 mortality (OR 1.86, 95%CI 0.88–3.94), similarly to the study by Karanasos et al. We also verified the statistically significant association between smoking and disease severity in the studies with low (< 15%) prevalence of diabetes, which was not observed in the studies with high diabetes prevalence (Fig. 4).

## Discussion

This re-analysis of the recently published study by Karanasos et al. identified a particularly low prevalence of smoking among hospitalized COVID-19. This is consistent with previous publications [5, 6, 46]. It should be emphasized that several limitations are applicable to this analysis, mainly related to the possibility for poor recording or under-reporting of the smoking status, lack of adjustment for confounding factors and potential differences in healthcare access between smokers and non-smokers. Another argument that has been suggested is that hospitalized COVID-19 cases are more likely to suffer from smoking-related comorbidities and might have already quit smoking because of these comorbidities. While this is a possibility, population surveys show that comorbidities, such as COPD, are still more prevalent in current rather than former smokers [47]. Some of the studies included in this analysis reported “smoking” only, without clarifying if former smokers where included in that group. Another type of selection bias could be linked to smokers being more likely to be tested for respiratory diseases than the general population. However, in a recent study of a large sample of COVID-19 patients in Mexico we noticed that smokers were less likely to be diagnosed for COVID-19, but the proportion of patients tested for COVID-19 who were smokers was not different from the population prevalence of smoking [48]. In any case, a vast difference between observed and expected, population-based, smoking prevalence was observed, even when the latter was adjusted by gender and age. The lack of high quality data or studies specifically evaluating the effect of smoking on COVID-19 susceptibility and severity is expected, considering the emergency of the pandemic. However, priorities, recommendations and treatment decisions should be based on best currently-available evidence, and any gaps in knowledge should be presented so that efforts to be resolved will be intensified.

Our analysis failed to reproduce the results of the study by Karanasos et al. concerning the association between smoking and COVID-19 severity. We consider the use of random-effects meta-analysis crucial when examining studies with such diverse and heterogeneous populations. However, other studies [2–4], including one by our group [46], found a positive association between smoking and adverse outcome among hospitalized COVID-19 patients. Still, choosing a proper methodology in any data analysis is important, irrespective of the study results or expectations. The low prevalence of smoking among hospitalized COVID-19 patients, combined with the increased odds for severe disease when smokers are hospitalized, which was not confirmed in this analysis but has been reported in other studies, should be accurately interpreted as a risk confined to a substantially smaller than the expected number of smokers who develop severe COVID-19 that requires hospitalization. This is different from interpreting the results as an indication of an elevated risk for all smokers. These findings are not contradictory to a hypothesis we recently presented that nicotine may have potential benefits, considering the rapid elimination of nicotine once smokers are admitted to the hospital and quit nicotine intake. In fact, clinicians should consider administering pharmaceutical nicotine replacement therapies in hospitalized smoking patients as “on-label” use, based on their indication as smoking substitutes [5]. Finally, while we verified the findings by Karanasos et al. concerning the studies with low prevalence of diabetes, the authors suggested that smoking may have a more pronounced adverse effect in younger, non-diabetic patients. However, this interpretation is problematic considering that their meta-regression is used to relate the results of the studies to published averages of patient characteristics within studies. This raises the possibility for ecological fallacy [49], which cannot be investigated due to the lack of individual patient data. There is no information in any of the studies on whether smokers were younger or had lower prevalence of diabetes compared to non-smokers. Thus, it is virtually impossible to generate such a conclusion from the meta-regression analysis.

Understandably, the issue of smoking is highly controversial and the findings of this and other studies may seem “paradoxical” and unexpected. Moreover, there may be concerns about the public perceiving smoking as a protective factor, which could discourage smoking cessation or might even encourage smoking relapse. However, it is the duty of the research community to focus on the data only and present them in an unbiased and balanced way, by emphasizing the limitations but also by avoiding potential predispositions or result expectations. In that respect, the well-established evidence on the adverse health effects of smoking raises no doubt that smoking initiation or continuation cannot be recommended as a protective measure for COVID-19 (or any other disease). However, the consistent data on low smoking prevalence among hospitalized patients, despite their limitations, raise the possibility nicotine could have potential therapeutic effects. Nicotine has been available for years in pharmaceutical formulations and has been used therapeutically even in non-smokers [50, 51]. It is also possible that other pharmaceutical agonists of nicotinic acetylcholine receptors may have similar therapeutic benefits [52], with animal studies showing effects similar to nicotine in promoting inflammatory control through the nicotinic cholinergic system [53–55]. Therefore, pharmaceutical nicotine replacement therapies or other nicotinic agonists should be investigated in experimental in vitro studies and in clinical trials as a potential therapeutic measure for COVID-19.

## Data Availability

The study presents an analysis of data presented in other studies**.**
